# Concurrent inhibition of mTORC1 and mTORC2 by WYE-687 inhibits renal cell carcinoma cell growth *in vitro* and *in vivo*

**DOI:** 10.1371/journal.pone.0172555

**Published:** 2017-03-03

**Authors:** Xiao-dong Pan, Dong-hua Gu, Jia-Hui Mao, Hua Zhu, Xinfeng Chen, Bing Zheng, Yuxi Shan

**Affiliations:** 1 The Department of Urology, The Second Affiliated Hospital of Soochow University, Suzhou, China; 2 Department of pathophysiology, Nantong University School of Medicine, Nantong, China; 3 The Department of Urology, The Second Affiliated Hospital of Nantong University, Nantong, China; Children's Hospital Boston, UNITED STATES

## Abstract

Mammalian target of rapamycin (mTOR)in renal cell carcinoma (RCC) represents a valuable oncotarget for treatment. We here tested the potential anti-RCC activity by a novel mTOR kinase inhibitor WYE-687*in vitro* and *in vivo*.WYE-687 was cytotoxic and anti-proliferative to established RCC cell lines (786-O and A498) and primary human RCC cells. Yet, it was non-cytotoxic toHK-2 tubular epithelial cells.WYE-687 provoked caspase-dependent apoptosis in the RCC cells. At the molecular level, WYE-687 almost completely blocked mTORC1 (p-S6K1 and p-S6) and mTORC2 (p-Akt Ser 473) activation in both 786-Ocells and primary human RCC cells, where it downregulated both hypoxia-inducible factor (HIF)-1α and HIF-2α expression. Significantly, oral administration of WYE-687 potently suppressed786-O tumor xenograft growth in nude mice. mTORC1/2 activation and HIF-1α/2α expression were also remarkably downregulated in WYE-687-treated tumor tissues. Thus, our preclinical results imply that WYE-687 may have important translational value for the treatment of RCC.

## 1. Introduction

Renal cell carcinoma (RCC) is a very common renal malignancy, which causes significant human mortalities each year [1,2,3,4]. The incidence of RCC has been rising in both Eastern and Western countries [1,2,3,4]. Epidemiological analysis has shown that RCCs are often diagnosed at advanced stages with local/systematic metastasis, and the prognosis and overall survival of these RCC patients are extremely poor [1,2,3,5,6]. The curable surgery resection of RCC is only available for patients with early-stage tumors and decent conditions[1,7,8,9]. Our lab [10,11,12] is dedicated to establishing valuable oncotargets for RCC, and to developing possible intervention strategies.

Existing evidences have reported mammalian target of rapamycin (mTOR) over-expression and/or hyper-activation in RCCs, which is associated with tumor progression [13]. mTOR is thus a valuable oncotarget for possible RCC treatment [13]. There are at least two multiple-protein mTOR complexes: the traditional mTOR complex 1 (mTORC1) and the late-identified mTOR complex 2 (mTORC2) [14,15]. The former, or mTORC1, is composed of mTOR, raptor, mLST8, and its activity can be inhibited by rapamycin and its analogs, or rapalogs[14,15]. mTORC1, by phosphorylatingp70S6K1 (S6K1) and eIF4E-binding protein 1 (4E-BP1), is important for a number of cancerous behaviors[14,15]. On the other hand, mTORC2 is assembled with mTOR, Rictor, Sin1 and mLST8, along with several others[14,15]. Literatures have demonstrated that mTORC2 shall function as the kinase of Akt and phosphorylate Akt at Ser-473 [14,15]. Both mTOR complexes are important for RCC cell progression and chemo-resistance [13,16,17,18].

Intriguingly, rapamycin and other rapalogs only displayed partial inhibition on mTORC1, and only inhibits mTORC2when given for prolonged periods of time[19]. Recently, the mTOR kinase inhibitors, or the second generation of mTOR inhibitors, were developed [19]. These inhibitors potently block both mTORC1 and mTORC2simantanuously[19]. Several of these mTOR kinase inhibitors are being tested in preclinical RCC models[18]. In the current study, we tested the potential anti-RCC activity by a novel mTOR kinase inhibitor WYE-687 [20], both *in vitro* and *in vivo*.

## 2. Material and methods

### 2.1. Reagents and chemicals

WYE-687, rapamycin and everolimus (RAD001) were purchased from Tocris Chemicals (Shanghai, China). The broad caspase inhibitor Ac-VAD-cho and the caspase-3 inhibitor Ac-DEVD-cho were obtained from Enzo Life Sciences (Shanghai, China). All the antibodies utilized in this study were described previously [10,11,12,21], and were purchased from Santa Cruz Biotechnology (Santa Cruz, CA) and Cell Signaling Technologies (Beverly, MA).

### 2.2. Culture of established cell lines

Human RCC cell lines (786-O and A489) as well as HK-2 tubule epithelial cells were obtained from the Cell Bank of Shanghai Institute of Biological Science (Shanghai, China). Regular 786-O cells were constructed with a wt-HIF-1α-expressing GV248 lentiviral vector (Genepharm, Shanghai, China) to establish the HIF-1α-expressing 786-O cells. The 786-O cells utilized in this study were then HIF-1α-postive. Every five months, DNA fingerprinting and profiling were performed to confirm the origin cell lines. The culture of established human RCC cell lines (786-O and A489) as well as HK-2 tubule epithelial cells was described in detail in our previous studies [10,11,12,21]. Cells were subjected to mycoplasma and microbial contamination examination every month. Population doubling time, colony forming efficiency, and morphology were also examined routinely.

### 2.3. Primary culture of human RCC cells

As described[12], tissue specimens were obtained from one RCC patient (Male, 54-year old) with total nephroureterectomy, who enrolled at The Second Affiliated Hospital of Nantong University (Nantong, China) and received no treatment prior to the surgery. The minced RCC tumor tissues were digested via collagenase I (Sigma, 0.05% w/v) incubation. Individual cells were pelleted, rinsed and filtered. Primary RCC cells were cultured in the FBS-DMEM/F12 medium, containing 10 ng/ml basic fibroblast growth factor (bFGF) and 10 ng/ml epidermal growth factor (EGF). Wells showing outgrowth of fibroblasts were omitted from further studies. Primary RCC cells of passage 3–6 were utilized for experiments. Experiments and the protocols in this study were approved by the Ethics Review Board (ERB) and Internal Review Board (IRB)of Nantong University (Nantong, China). The written-informed consent was obtained from the enrolled patient. All investigations were conducted according to the principles expressed in the Declaration of Helsinki as well as national/international regulations.

### 2.4. Methylthiazol tetrazolium (MTT) assay

After applied treatment, cell survival was assessed through routine MTT assay. The detailed protocol was described early[10,11,12,21].

### 2.5. Clonogenicity assay

The protocol was described in our previous studies[10,11,12,21]. Briefly, the 786-O cells were treated with WYE-687 every 2days for a total of 10 days. Afterwards, the number of viable colonies, stained with crystal blue, were counted manually.

### 2.6. Annexin V assay of cell apoptosis

As reported [22], following the applied treatment, cells were washed and incubated with Annexin V-FITC and propidium iodide (PI) (Invitrogen, Shanghai, China). Afterwards, cells were then detected through fluorescence-activated cell sorting (FACS) with a Becton-Dickinson machine (San Jose, CA). Annexin V-stained cells were gated as the apoptotic cells, and its ratio was recorded.

### 2.7. Caspase-3 activity assay

Assaying of caspase-3 activity was described early[11]. For each treatment, 10 μg of cytosolic extracts were added to caspase assay buffer [11] with the caspase-3 substrate [11]. Release of 7-amido-4-(trifluoromethyl)-coumarin (AFC) was quantified via a Fluoroskan system [11]. TheAFC optic density (OD) of treatment group was normalized to that of untreated control group.

### 2.8. [H^3^] Thymidine incorporation assay

As described [23], RCC cells (1 × 10^5^ cells/well) were seeded onto the 48-well plates. Cells were treated with applied WYE-687 in the presence of thymidine (1 μCi/mL, Sigma). Afterwards, cells were washed, and the cellular DNA was precipitated [24]. The aliquots were counted by liquid-scintillation spectrometry. The [H^3^] Thymidine value of treatment group was normalized to the control value.

### 2.9. BrdU assay

Following applied treatment, cells were incubated with BrdU (10 μM, Cell Signaling Tech, Shanghai, China), and were fixed. BrdU incorporation was determined in the ELISA format according to the protocol. BrdU OD value of treatment group was normalized to that of untreated control group.

### 2.10. Western blot assay

As described[10,11,12,21],cell and tumor tissue lysates were fractionated on SDS-page gels, and were transferred to nitrocellulose blots. The blots were probed with a designated primary antibody, followed by incubation of the corresponding second antibody (Pierce). Enhanced chemiluminescence (ECL) reagents(GE Healthcare, Shanghai, China) were applied to visualize the interested bands.

### 2.11. Xenograft model

Female nude/beige mice, 4–5 week old, 16–18g, were purchased from Nantong University Animal Laboratories (Nantong, China). 786-O RCC cells (5 × 10^6^per mouse) were injected into left flanks. Within three week, the tumor xenografts were established, and the tumor volumes were around 100 mm^3^. Mice (n = 10 each group) were treated once daily by gavage with either vehicle control or WYE-687 (25 mg/kg body weight) for 15 consecutive days[20,24]. Mice body weight and bi-dimensional tumor measurements were taken every 5 days. Tumor volume was calculated as described [11]. Mice in this study were observed on a daily basis very closely. Humane endpoints were considered as rapid weight loss (>10%), severe fever, vomiting or skin problems (wounds or signs of inflammation). If animals reached these endpoints, they were euthanized by exsanguination under 2,2,2-tribromoethanol anesthesia (4 mg/10 g body weight, Sigma). All injections in this study were performed via the 2,2,2-tribromoethanol anesthesia method. The animal protocol was approved by the Institutional Animal Care and Use Committee (IACUC) and Ethics Review Board (ERB) of Nantong University (Nantong, China).

### 2.12. Immunohistochemistry (IHC) staining

The IHC staining was performed on cryostat sections (4 μm/section) of 786-O xenograft tumors, and the protocol was described in detail in previous studies [12,25]. The primary antibody (anti-Akt Ser-473, 1: 50, Cellular Signaling Tech), and horseradish peroxidase (HRP)-coupled secondary antibody (Santa Cruz) were applied [12]. The positive staining was performed through peroxidase activity using the 3-amino-9-ethyl-carbazol (AEC) method (Merck, Shanghai, China).

### 2.13. Statistical analysis

Data were expressed as mean ± standard deviation (SD). The data in each figure were summarizing one set of experiment. The whole set of experiments were always repeated 3–5 times of each figure (expect for the *in vivo* experiments), and similar results were obtained. Statistical analyses were performed by one-way analysis of variance (ANOVA) with the GraphPad software. IC-50 was calculated by the SPSS 17.0 software. Significance was set at p < 0.05.

## 3. Results

### 3.1. WYE-687 is cytotoxic to cultured human RCC cells

**Fig 1:**
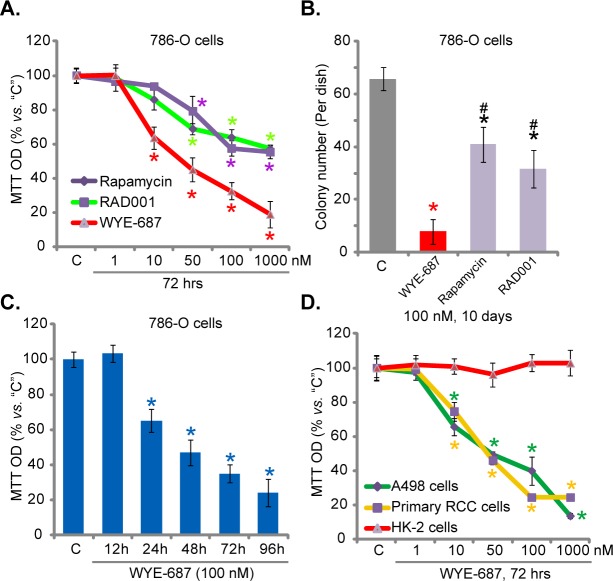
WYE-687 is cytotoxic to cultured human RCC cells. Established human RCC cell lines (786-O and A498), primary human RCC cells, or HK-2 tubular epithelial cells were treated with indicated concentration of WYE-687, rapamycin or RAD001 for applied time, cell viability was tested by MTT assay (A, C and D, n = 5). 786-O cells were treated with 100 nM of WYE-687, rapamycin or RAD001 for 10 days, the number surviving colonies was recorded (B, n = 5). *p< 0.05 vs. untreated control group (“C”). ^#^*p*< 0.05 vs. WYE-687 group (B).Vehicle control (0.1% of DMSO) failed to affect survival and proliferation of above cells.

In order to test the potential activity of WYE-687 in RCC cells, 786-O RCC cells, cultured in FBS-containing complete medium, were treated with gradually increasing concentrations (from 1 nM to 1000 nM) of the mTOR kinase inhibitor. MTT viability assay results in Fig 1A demonstrated that WYE-687 dose-dependently reduced786-O cell survival, and the MTT OD of 786-O cells was significantly decreasedafter10-1000 nM of WYE-687 treatment (Fig 1A). The WYE-687’s IC-50, or the concentration that inhibited 50% of cell survival, was 23.21± 2.25 nM (Fig 1A). Remarkably, the anti-survival activity of WYE-687 was significantly more potent than the same concentration of rapamycin and RAD001, two knownmTORC1 inhibitors (Fig 1A) [26,27].For example, at 50 nM, WYE-687 led to about 55% of 786-O cell viability reduction, yet same concentration of rapamycin and RAD001 only induced ~20% and 31% of viability reduction, respectively (Fig 1A). The IC-50s for rapamycin and RAD001 were both over 1000 nM (Fig 1A). Clonogenicity assay results in Fig 1B demonstrated that WYE-687 (100 nM) treatment dramatically reduced the number of viable 786-O colonies. Its activity was again significantly more potent than same concentration of rapamycin and RAD001 (Fig 1B). Results in Fig 1C demonstrated a time-dependent response by WYE-687 (100 nM) in inhibiting786-O cell survival. It took only 24 hours for the mTOR kinase inhibitor to exert a significant anti-survival activity (Fig 1C).

We also tested the activity of WYE-687 on other RCC cells. In both A498 cells, an established RCC cell line[28,29], and primary human RCC cells, treatment with WYE-687 again dose-dependently decreased cell survival MTT OD (Fig 1D). WYE-687 was again efficient in inhibiting these RCC cells, with IC-50 less than 50 nM for both cell lines (Fig 1D).Remarkably, the very same WYE-687 treatment failed to significantly affect the survival of HK-2 cells (Fig 1D), which are normal tubular epithelial cells[30,31]. Together, these results are consistent with the hypothesis thatWYE-687 is cytotoxic to cultured human RCC cells.

### 3.2. WYE-687 induces apoptosis in cultured human RCC cells

**Fig 2:**
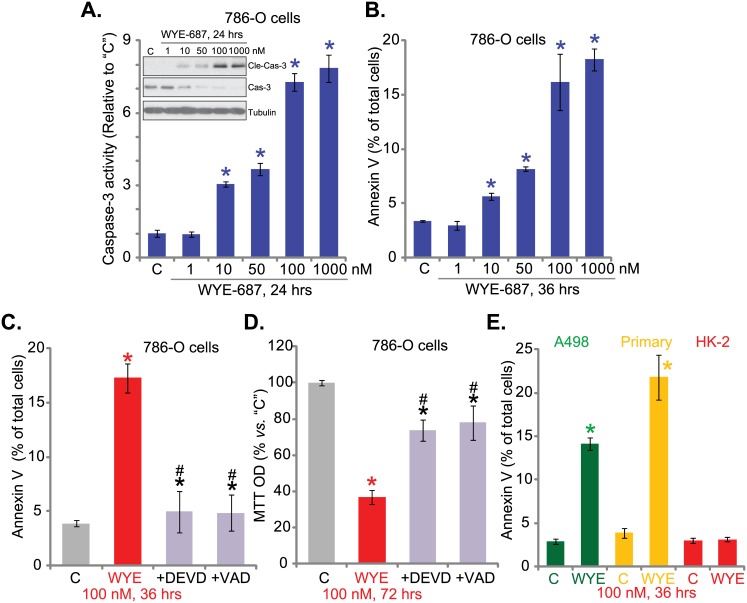
WYE-687 induces apoptosis in cultured human RCC cells. RCC cell lines (786-O and A498), primary human RCC cells (“Primary”), or HK-2 normal tubular epithelial cells were treated with described WYE-687 (“WYE”) for applied time, cell apoptosis was tested by listed assays (A, B, E, n = 3). For C and D, 786-O cells were pre-treated for 1 hour with Ac-DEVD-cho (“+DEVD”, 50 μM) or Ac-VAD-cho (“+ VAD”, 50 μM) before applied WYE-687 treatment, cell apoptosis (Annexin V assay, C, n = 3) and survival (MTT assay, D, n = 5) were tested. *p< 0.05 vs. untreated control group (“C”). ^#^*p*< 0.05 vs. WYE-687treatment only (C and D).

Next, we tested the potential effect of WYE-687 on cell apoptosis. In line with our previous studies[10,11,12], cell apoptosis was tested by caspase-3 activity assay and Annexin VFACS assay. Results from both assays demonstrated that WYE-687 dose-dependently induced 786-O cell apoptosis (Fig 2A and 2B). The caspase-3 activity (Fig 2A) and the number of cells with Annexin V staining (Fig 2B) were both significantly increased following 10–1000 nM of WYE-687 treatment. Meanwhile, caspase-3 cleavage(“Cle-Cas-3”) was induced by WYE-687 treatment in 786-O cells (Fig 2A, upper panel).Notably, as shown in our previous study[11], rapamycin and RAD001 failed to induce significant apoptosis in 786-O cells. Significantly, WYE-687 (100 nM)-induced 786-O cell apoptosis and the Annexin V staining increased, an increase which was largely inhibited by either the caspase-3 inhibitor Ac-DEVD-cho or the pan caspase inhibitor Ac-VAD-cho (Fig 2C). Meanwhile, the two caspase inhibitors significantly attenuated the WYE-687-induced reduction in 786-O cell viability (Fig 2D).Annexin V assay results in Fig 2E showed that WYE-687 (100 nM, 36 hours) similarly induced profound apoptosis in A498 RCC cells and primary human RCC cells. Yet, no significant apoptosis was observed in WYE-687-treated HK-2 tubular epithelial cells (Fig 2E). Together, these results suggest that WYE-687 provokes caspase-dependent apoptosis in RCC cells.

### 3.3. WYE-687 inhibits human RCC cell proliferation

**Fig 3:**
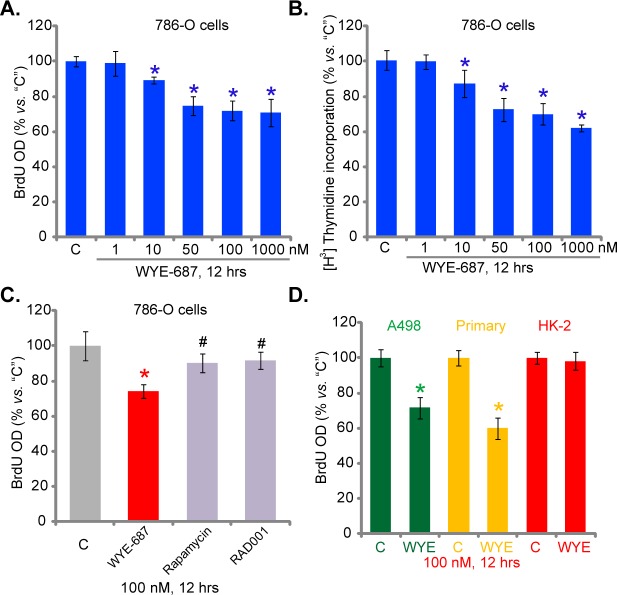
WYE-687 inhibits human RCC cell proliferation. RCC cell lines (786-O and A498), primary human RCC cells, or HK-2 tubular epithelial cells were treated with indicated concentrations of WYE-687, rapamycin or RAD001 for applied time, cell proliferation was tested by listed assays (A-D, n = 5). *p< 0.05 vs. untreated control group (“C”). ^**#**^*p*< 0.05 vs. WYE-687 group (C).

Next, we tested the effect of WYE-687 on RCC cell proliferation. Two well-established proliferation assays, including the [H^3^] Thymidine incorporation assay and BrdU incorporation ELISA assay [23,32]were performed. Results from both assays demonstrated that WYE-687 dose-dependently inhibited 786-O cell proliferation (Fig 3A and 3B). The BrdU ELISA OD (Fig 3A) and [H^3^] Thymidine incorporation(Fig 3B) were both significantly decreased following WYE-687 (10–1000 nM) treatment. Once again, WYE-687 was more efficient than rapamycin and RAD001 in inhibiting786-O cell proliferation (Fig 3C). BrdU ELISA assay results in Fig 3D confirmed that WYE-687 (100 nM) was also anti-proliferative againstA498 RCC cells and primary human RCC cells. On the other hand, the proliferation of HK-2 tubular epithelial cells was again not altered following the WYE-687 treatment (Fig 3D). Notably, for testing cell proliferation, RCC cells were treated with WYE-687 for only 12 hours, when no significant cytotoxicity was yet noticed (Fig 1C). Collectively, these results show that WYE-687 inhibits RCC cell proliferation.

### 3.4. WYE-687 blocks mTORC1 and mTORC2 activation in RCC cells

**Fig 4:**
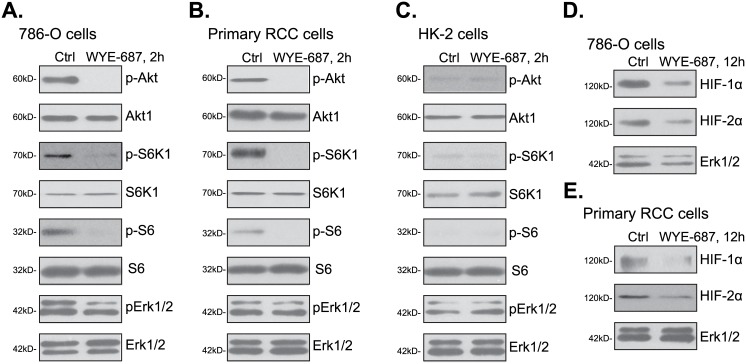
WYE-687 blocks mTORC1 and mTORC2 activation in RCC cells. 786-O cells (A and D), primary human RCC cells (B and E), or HK-2 tubular epithelial cells (C) were treated withWYE-687 (100 nM) for applied time, expression of listed proteins was tested by Western blot assay.

Since WYE-687 is a novel mTOR kinase inhibitor[20,24], its effect on mTOR signaling was tested. As shown in Fig 4A, treatment with WYE-687 (100 nM, 2 hours) in 786-ORCC cells almost completely blocked phosphorylation (“p-”) of Akt (Ser-473), S6K1 (Thr-389) and S6 (Ser-235/236). These results indicated that WYE-687 blocked both mTORC1 (indicated by p-S6K1, p-S6[15]) and mTORC2 (indicated by p-Akt Ser 473[15]) in 786-O cells. Expression of the total proteins was not affected by the WYE-687 treatment (Fig 4A). Very similar results were also observed in the primary human RCC cells, where WYE-687 (100 nM, 2 hours) treatment blocked mTORC1 (p-S6K1, p-S6) and mTORC2 (p-Akt Ser 473)activation simantanuously (Fig 4B). In the HK-2 tubular epithelial cells, the basal activation of mTORC1 (p-S6K1, p-S6) and mTORC2 (p-Akt Ser 473) was extremely low, as compared to the above RCC cells (Fig 4C). This might explain why these cells were not killed by the mTOR kinase inhibitor (Figs 1 and 2). Intriguingly, Erk-MAPK activation, tested by p-Erk1/2 (Thr202/Tyr204), was not significantly changed following WYE-687 treatment (Fig 4A–4C).Groups including ours[10,11,12] have confirmed that activation of mTOR is important for the expression of both HIF-1α and HIF-2α. Therefore, WYE-687 shall affect expressionofHIF-1α and HIF-2α in HCC cells. Indeed, in both 786-O cells (Fig 4D) and primary human RCC cells (Fig 4E), WYE-687 (100 nM, 12 hours) treatment induced significant downregulation of HIF-1α and HIF-2α. Together, we show that WYE-687 in-activates mTORC1 and mTORC2, and depletes HIF-1α and HIF-2α in human RCC cells.

### 3.5. WYE-687 oral administration inhibits 786-O RCC tumor growth in nude mice

**Fig 5:**
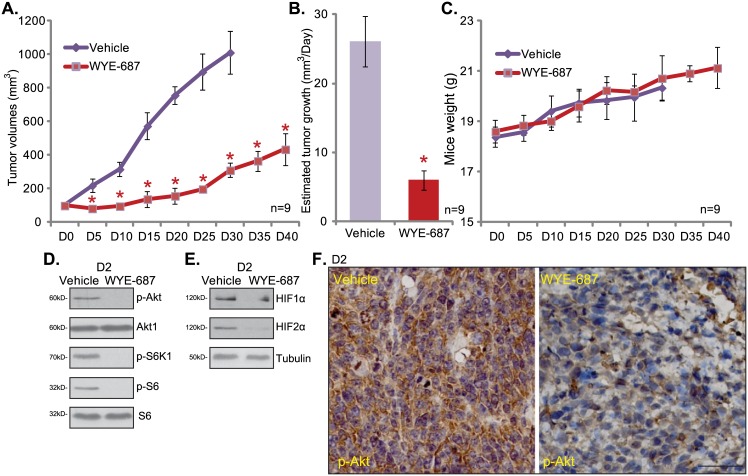
WYE-687 oral administration inhibits 786-O RCC tumor growth in nude mice. The growth curve of 786-O xenografts in nude beige mice with daily administration ofWYE-687 (oral gavage, 25 mg/kg body weight) or vehicle control (“Vehicle”) was presented (A). Each treatment group comprised 9 mice, mean estimated tumor volume (A) and mice body weight (C) were recorded every 5 days. Estimated daily tumor growth was also presented (B). To test signaling changes, at treatment day-2, one mice per group was sacrificed, and tumor xenografts were excised; Expressions of indicated proteins in xenograft tissues were analyzed by Western blot assay (D and E) and IHC staining assay (F, bar = 50 μm). *p< 0.05.

The potential anti-RCC activity of WYE-687 *in vivo* was also tested. As described[11], the786-ORCC tumor xenograft model was applied. A significant number of 786-O cells were inoculated into the nude mice[11].Within three weeks, the xenograft RCC tumors were established with the average tumor volumes of 100 mm^3^. Half of the mice were treated with WYE-687 (25 mg/kg body weight, oral gavage, daily, for 15 days)[20,24]. The other half mice were administrated with vehicle control (5% ethanol, 2% Tween 80, and 5% polyethylene glycol-400) [24].As demonstrated in Fig 5A, 786-O tumor growth in the WYE-687-administrated mice was significantly slower than that of vehicle control mice. The WYE-687-treated tumors were much smaller than the vehicle-treated tumors (Fig 5A). Results in Fig 5B demonstrated that, with WYE-687 administration, the estimated tumor growth (mm^3^ per day) was significantly lower. Notably, WYE-687-treated mice didn’t present any signs of wasting, and the mice body weight was not different from that of vehicle-treated mice (Fig 5C). We also failed to notice any apparent toxicities (vomiting, fever, diarrhea) in the tested mice.

mTOR signaling in WYE-687-treated tumors was also tested. At day-2 after initial WYE-687 treatment, one xenograft tumor of each group was isolated. Western blot results assaying of tumor lysates showed that activation of mTORC1 (p-S6K1, p-S6) and mTORC2 (p-Akt Ser 473)was largely inhibited in WYE-687-treated tumors. Meanwhile, expression of HIF-1α and HIF-2α was also significantly decreasedafterWYE-687 administration (Fig 5E). Immunohistochemistry (IHC) images in Fig 5F further confirmed p-Akt Ser 473 silence in the 786-O tumor with WYE-687 administration. Therefore, WYE-687 oral administration also in-activates mTORC1/mTORC2, and depletes HIF-1α/HIF-2α *in vivo*.

## 4. Discussions

Rapamycin and its analogs (rapalogs, *i*.*e*.RAD001, CCI-779, AP23573) are mTORC1 inhibitors[33]. The clinical use of these mTORC1 inhibitors could have several drawbacks. Thus, the anti-cancer efficiency of these agents is often moderate, even combined with the conventional chemo-agents [19,34]. It is now known that rapalogs only partially inhibit 4E-BP1 phosphorylation, leading to incomplete inhibition of mTORC1 [19,34]. Rapamycin and its analogs could only inhibit mTORC2 when given at high doses for prolonged periods of time [35,36]. Also their inhibition on mTORC2 is certainly weak than mTOR kinase inhibitors [37].Meanwhile, treatment of rapalogs shall provoke feedback activation of several pro-cancerous cascades, *i*.*e*. Akt and Erk-MAPK. Activation of these signalings would counteract their anti-cancer activity [19,34]. Further, water solubility of these rapalogs is often extremely poor [19,34].

Therefore, mTOR kinase inhibitors, or second generation of mTOR inhibitors, were developed [19,34]. These mTOR kinase inhibitors shall block mTORC1 and mTORC2 simantanuously, without leading to feedback activation of above oncogenic signalings[19,34]. Many of these mTOR kinase inhibitors have displayed superior anti-tumor efficiency in preclinical cancer researches[19,34]. Here, we found that WYE-687, a novel mTOR kinase inhibitor [20,24], simantanuously blocked mTORC1 and mTORC2 activation in established and primary human RCC cells. Significantly, WYE-687 was remarkably more potent than the rapalogs (rapamycin and RAD001) in inhibiting RCC cell survival and proliferation. Thus, the advantage of using this novel mTOR kinase inhibitor to kill RCC cells is obviously very striking. Not to mention it exerted no significant cytotoxicity to non-cancerous HK-2 tubular epithelial cells.To our best knowledge, this is the first report testing WYE-687’s activity in RCC cells.

pVHL (von Hippel–Lindau protein) is the E3 ubiquitin ligase for HIF-1α/2α degradation [38]. pVHL inactivation or mutation will lead to HIF-1α/2α stabilization and accumulation, causing transcription of vascular endothelial growth factor (VEGF) and other oncogenic proteins[38]. A large proportion (around 50%) of RCC patients have pVHL mutations[38]. Intriguingly, it has been proposed that HIF-2α is far more important than HIF-1α in RCC tumorigenesis and progression [39]. As a matter of fact, silence of HIF-2α, but not HIF-1α, could abolish tumorigenesis of pVHL-depleted RCC [39]. pVHL-mediated tumor suppression is also nullified with forced-expression of HIF-2α (but not HIF-1α) [40].

Interestingly, recent studies have shown that mTORC2, but not mTORC1, dictates HIF-2α translation [41]. And mTORC1 is the major upstream signaling for HIF-1α expression[41].In this study, we show that WYE-687 concurrently blocked mTORC1 and mTORC2 activation, consequently leading to HIF-2α and HIF-1α depletion *in vitro* and *in vivo*. These results are consistent with our previous findings[11],showing that mTORC1 and mTORC2 blockage by AZD-2014 downregulated both HIF-1α and HIF-2αin RCC cells. Cho *et al*., similarly showed that NVP-BEZ235, a dual PI3K/mTOR kinase blocker, induced profound HIF-2α degradation [29]. Obviously,WYE-687’s ability in downregulating HIF-1/2α was far more efficient that rapalogs. Our previous studies have shown that RAD001 had almost no effect on HIF-2α, and only induced moderate HIF-1α downregulation [11]. Other studies have shown that rapamycin was unable to affect HIF-2α expression [29]. Thus, concurrent blockage of mTORC1 and mTORC2 by WYE-687 shall downregulate HIF-1α and HIF-2α in RCC cells.

## 5. Conclusions

In conclusion, we show that WYE-687 concurrently blocks mTORC1 and mTORC2, and inhibits RCC cell growth both *in vitro* and *in vivo*. Based on these results, we imply that concurrent blockage of mTORC1 and mTORC2 should be the reason of the superior anti-RCC activity by WYE-687. Future studies will also be needed to further confirm this hypothesis. Everolimus and other rapamycin analogs are approved by FDA for treatment of RCC clinically[13,17]. These rapalogs have displayed fine clinical benefits for RCC patients [13,17]. Our results showing WYE-687 was significantly more potent than rapalogs in inhibiting RCC cells suggesting that WYE-687 might possibly be an important improvement of rapalogs for RCC treatment.
